# Different effects of monophasic pulses and biphasic pulses applied by a bipolar stimulation electrode in the rat hippocampal CA1 region

**DOI:** 10.1186/s12938-021-00862-y

**Published:** 2021-03-09

**Authors:** Yue Yuan, Lvpiao Zheng, Zhouyan Feng, Gangsheng Yang

**Affiliations:** Key Laboratory of Biomedical Engineering of Education Ministry, College of Biomedical Engineering and Instrumentation Science, Zhejiang University, Hangzhou, 310027 Zhejiang China

**Keywords:** High-frequency stimulation, Monophasic pulse, Biphasic pulse, Spreading depression, Hippocampal CA1 region

## Abstract

**Background:**

Electrical pulse stimulations have been applied in brain for treating certain diseases such as movement disorders. High-frequency stimulations (HFS) of biphasic pulses have been used in clinic stimulations, such as deep brain stimulation (DBS), to minimize the risk of tissue damages caused by the electrical stimulations. However, HFS sequences of monophasic pulses have often been used in animal experiments for studying neuronal responses to the stimulations. It is not clear yet what the differences of the neuronal responses to the HFS of monophasic pulses from the HFS of biphasic pulses are.

**Methods:**

To investigate the neuronal responses to the two types of pulses, orthodromic-HFS (O-HFS) and antidromic-HFS (A-HFS) of biphasic and monophasic pulses (1-min) were delivered by bipolar electrodes, respectively, to the Schaffer collaterals (i.e., afferent fibers) and the alveus fibers (i.e., efferent fibers) of the rat hippocampal CA1 region in vivo. Evoked population spikes of CA1 pyramidal neurons to the HFSs were recorded in the CA1 region. In addition, single pulses of antidromic- and orthodromic-test stimuli were applied before and after HFSs to evaluate the baseline and the recovery of neuronal activity, respectively.

**Results:**

Spreading depression (SD) appeared during sequences of 200-Hz monophasic O-HFS with a high incidence (4/5), but did not appear during corresponding 200-Hz biphasic O-HFS (0/6). A preceding burst of population spikes appeared before the SD waveforms. Then, the SD propagated slowly, silenced neuronal firing temporarily and resulted in partial recovery of orthodromically evoked population spikes (OPS) after the end of O-HFS. No SD events appeared during the O-HFS with a lower frequency of 100 Hz of monophasic or biphasic pulses (0/5 and 0/6, respectively), neither during the A-HFS of 200-Hz pulses (0/9). The antidromically evoked population spikes (APS) after 200-Hz biphasic A-HFS recovered to baseline level within ~ 2 min. However, the APS only recovered partially after the 200-Hz A-HFS of monophasic pulses.

**Conclusions:**

The O-HFS with a higher frequency of monophasic pulses can induce the abnormal neuron activity of SD and the A-HFS of monophasic pulses can cause a persisting attenuation of neuronal excitability, indicating neuronal damages caused by monophasic stimulations in brain tissues. The results provide guidance for proper stimulation protocols in clinic and animal experiments.

## Background

Electrical pulses have been used in deep brain stimulation (DBS) for treating certain neurological disorders successfully (e.g., Parkinson's disease and epilepsy) and for treating psychiatric disorders promisingly (e.g., major depression and obsessive compulsive disorder) [1, 2]. The DBS commonly utilizes sequences of narrow pulses with a pulse width around 0.1 ms and a pulse frequency ~ 100–200 Hz (termed as high-frequency stimulation, HFS). Charge-balanced biphasic pulses (with a preceding negative pulse immediately followed by a positive one) are usually utilized in clinical DBS for safety [3, 4], while negative monophasic pulses are used in animal studies for investigating brain stimulations [5–7]. However, it is not clear whether HFS sequences of monophasic pulses could induce abnormal neuronal responses different from biphasic pulses.

In theory, a negative pulse is more efficient to activate neurons than a positive pulse or a biphasic pulse in the situation of extracellular stimulation such as DBS [8, 9]. However, a continuous stimulation of a sequence of monophasic pulses could risk tissue damages caused by irreversible chemical reactions, whereas biphasic pulses could not [8, 10]. The reverse electric field generated by the subsequent positive phase of a biphasic pulse may prevent an accumulation of cation/anion ions thereby preventing irreversible chemical reactions in the brain tissue around the electrode contacts. However, sequences of monophasic negative pulses could result in irreversible chemical reactions to generate toxic products to damage the brain tissue [8].

Besides the toxic products of electrochemical reactions, stimulation-related tissue damages may be also caused by other factors, such as depletion of oxygen by hyperactivity of neuronal firing, substantial changes of ionic concentrations in both intracellular and extracellular environments, and excitotoxicity caused by excessive release of glutamates [8, 11]. Some of the changes may lead to abnormal neuronal activities, such as a large shift of depolarization potential in brain regions, termed as spreading depression (SD) [12]. To investigate the differences of neuronal responses to HFS of monophasic pulses and biphasic pulses, we recorded the electrical signals in the hippocampal CA1 region in rat brain in vivo during HFS of axonal fibers and observed the neuronal responses.

The hippocampal region of brain is prone to generate SD events under certain circumstances, such as an increase of extracellular potassium [13, 14]. In addition, axons are the most susceptible structure of neurons to the extracellular stimulation of narrow electrical pulses [15, 16]. Therefore, the incidence of SD induced by axonal HFS in hippocampal CA1 region could be used as a biomarker to distinguish the neuronal responses to different sequences of HFS. Results of the study may provide new information for appropriate usage of pulse stimulations in the investigations and in the clinical applications of brain stimulations.

## Results

### Spreading depression appeared during orthodromic-HFS with monophasic but not biphasic pulses

**Fig. 1:**
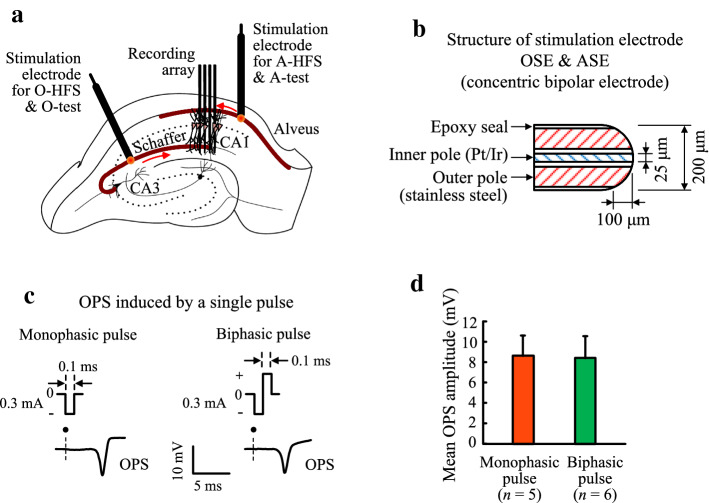
Schematic diagram of electrode locations, electrode geometry and waveforms of evoked potentials. **a** The recording array in the hippocampal CA1 region, and stimulation electrodes for orthodromic activation and antidromic activation in the Schaffer collaterals and alveus fibers, respectively. **b** The tip structure of the concentric bipolar stimulation electrodes. **c** Examples of orthodromically evoked population spike (OPS) evoked by a single monophasic and biphasic pulse. **d** Comparison of mean amplitudes of baseline OPS induced by single pulses of monophase (*n* = 5) and biphase (*n* = 6) with a same current intensity

To investigate the different effects between biphasic and monophasic pulses, HFS sequences of pulses were delivered through the stimulation electrodes placed at the Schaffer collaterals for the orthodromic-HFS (O-HFS) and at the alveus fibers for the antidromic-HFS (A-HFS), respectively (Fig. 1a). The stimulation electrodes were a concentric bipolar structure (Fig. 1b). For the stimulations of a single pulse with an identical current intensity, the mean amplitude of orthodromically evoked population spike (OPS) induced by a monophasic pulse (8.62 ± 1.97 mV, *n* = 5) was not significantly different from that induced by a biphasic pulse (8.40 ± 2.16 mV, *n* = 6; *t*-test, *P* = 0.95. Fig. 1c and d), which indicated a similar excitation action of the two types of pulses.

**Fig. 2:**
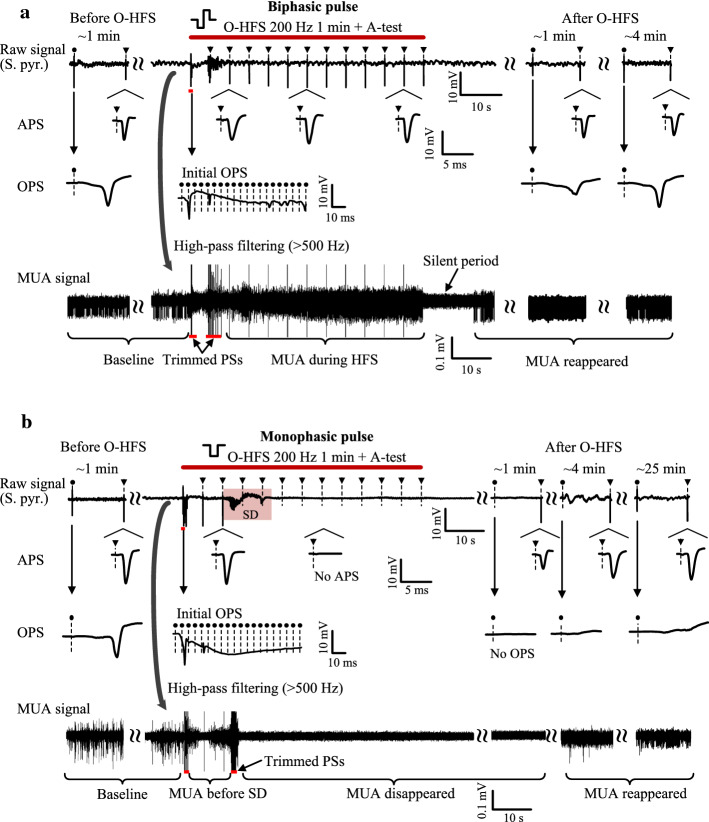
Spreading depression (SD) induced by 200-Hz O-HFS of monophasic pulses in the rat hippocampal CA1 region. **a** and **b** Typical examples of recording signals in the CA1 pyramidal layer with 1-min 200-Hz O-HFS (denoted by the red bar) of biphasic pulses (**a**) or monophasic pulses (**b**). The pink shade in **b** denotes an SD event. The APS and OPS waveforms induced by A-test pulses at the alveus and by O-test pulses at the Schaffer collaterals in the periods before, during and after O-HFS are expanded. The small inverted triangle with a dotted line denotes an A-test pulse and the dot with a dotted line denotes an O-test pulse. The MUA signals in the periods before, during and after O-HFS were obtained by filtering the raw signals

During the O-HFS of 1-min 200 Hz of biphasic pulses, OPS events only appeared in the initial several seconds of O-HFS (Fig. 2a). After the disappearance of OPS, multiple unit activity (MUA) continued to the end of the O-HFS with a firing rate of unit spikes higher than baseline. A silent period (10–30 s) without MUA appeared immediately following the end of O-HFS, indicating that the unit spikes during the late O-HFS period were induced by the stimulation. During the period of O-HFS, an antidromic-test (A-test) pulse was applied every 5 s (i.e., 0.2 Hz) at the alveus fibers to evaluate the excitability of the CA1 neurons. The single A-test pulses and orthodromic-test (O-test) pulses were also applied before and after O-HFS to evaluate the baseline and the recovery state of neuronal activity, respectively. Large antidromically evoked population spike (APS) evoked by A-test pulses persisted throughout the 1-min O-HFS, and the mean amplitude of these APS (7.26 ± 5.59 mV, *n* = 6) was ~ 17% greater than the corresponding baseline level (6.19 ± 3.19 mV, *n* = 6; paired *t*-test, *P* < 0.05). The results indicated that the sustained O-HFS increased the excitability of the CA1 neurons. About 4 min after the end of O-HFS, both test APS and test OPS evoked by single pulses recovered to the baseline level. In addition, no SD event appeared in all of the 6 rats with the 200-Hz biphasic O-HFS.

However, SD events appeared in 4 of the 5 rats with the 200-Hz monophasic O-HFS (Fig. 2b). The initial neuronal responses induced by the monophasic O-HFS was similar to that induced by the biphasic O-HFS: large OPS appeared at first, then OPS disappeared and dense MUA appeared. However, an SD appeared later with a slow waveform lasting 4.31 ± 3.11 s (*n* = 4). At the same time, the MUA disappeared completely and the A-test pulses were no longer able to induce an APS, indicating a silence of neuronal activity. The MUA did not appear until 3.51 ± 2.47 min (*n* = 4) after the end of O-HFS (Fig. 2b, bottom). By this time, the amplitude of test APS recovered to ~ 80% of the baseline level. However, the test OPS did not recover even ~ 25 min after the end of O-HFS, while the test APS had almost recovered to baseline level (89.5 ± 9.7%, *n* = 4. Fig. 2b, middle). The results indicated that 1-min persisted 200 Hz of monophasic O-HFS generated abnormal reactions of the neuronal population.

**Fig. 3:**
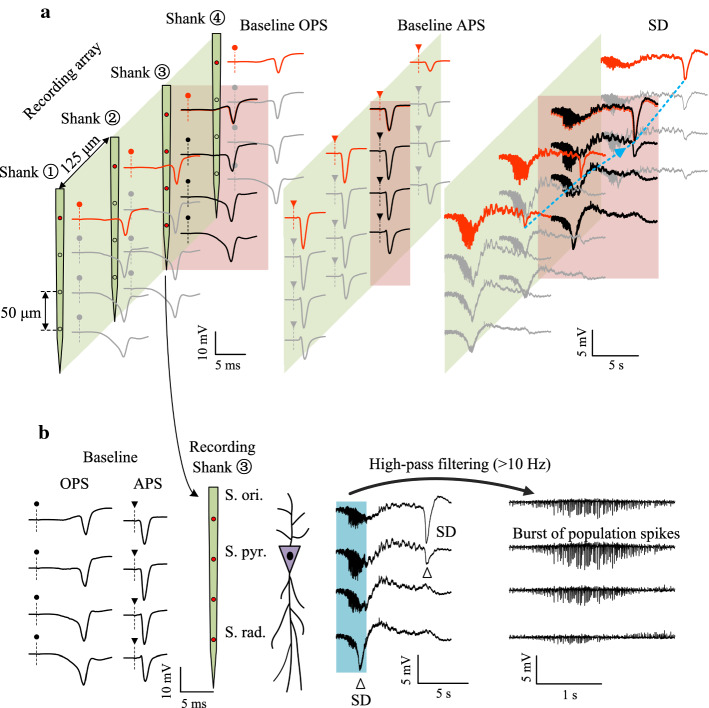
Propagation of an SD waveform. **a** Signals recorded by the four parallel shanks of the 16-channel recording array. Left and middle: baseline OPS and APS waveforms evoked by a single pulse. Right: an SD event in the 16-channel recordings. The blue dotted line denotes the movement of the SD trough in the horizontal direction. **b** The 4-channels of recording signals along the shank of the recording array. The waveforms of baseline OPS and APS indicate the locations of each recording channel in the different stratums of CA1 region. The hollow triangles denote the movement of the SD trough in the perpendicular direction. The blue shade denotes the burst of population spikes at the onset of SD that are filtered and expanded on the right

To show the spread of SD waveforms, we used the recording electrode array with four shanks and total 16 contacts (Figs. 1a and 3a). The waveforms of OPS and APS along the shanks in baseline recordings indicated the locations of each recording contact in the different stratums of CA1 region [17]. Because the signal recordings in the study were AC-coupled (0.3–5000 Hz), the SD waveform appeared as a trough similar to previous reports [18]. The SD trough appeared first in the stratum radiatum (S. rad.) of hippocampal CA1 region (Fig. 3b) and accompanied by a burst of population spikes (60–80 spikes/s) in the stratum pyramidale (S. pyr.). The burst was obvious in the filtered signals greater than 10 Hz (Fig. 3b right). Then, the SD trough propagated slowly to the CA1 layers of S. pyr. and stratum oriens (S. ori.) at a speed of 90 ± 51 μm/s (*n* = 4) in the perpendicular direction, characterized by the movement of the negative peak of SD trough along the recording shanks (Fig. 3b, hollow triangles). Also, the SD trough moved at a speed of 826 ± 627 μm/s (*n* = 4) in the S. pyr. layer transversely among the recording shanks (Fig. 3a right, blue dotted line). The characteristics of the SD events, including the waveform, the accompanied burst of population spikes, the slow travelling speed and the silence of neuronal electrical activity, were consistent with previous reports [18, 19].

The statistical results showed that the SD incidence during 200-Hz monophasic O-HFS (4/5) was significantly greater than the incidence during 200-Hz biphasic O-HFS (0/6; Fisher’s exact test, *P* < 0.05). In addition, with a decrease of the O-HFS frequency from 200 to 100 Hz, no SD events were observed with monophasic O-HFS (five rats) or with biphasic O-HFS (six rats). Therefore, for the data of monophasic O-HFS only, the SD incidence during 100-Hz O-HFS (0/5) was significantly lower than that during 200-Hz O-HFS (4/5; Fisher’s exact test, *P* < 0.05).

These results indicated that O-HFS of monophasic pulses with a higher stimulation frequency can generate SD events in the hippocampal CA1 region and affect the orthodromic pathway persistently. Because the generation of OPS by the stimulation at afferent fibers involves both the axonal conductions and the synaptic transmissions, the non-recovery of OPS after the monophasic O-HFS could have been caused by potential damages in the axons and/or synapses. To verify whether the monophasic HFS could cause damages in axons, we next inspected the responses of CA1 neurons to the A-HFS at their own axons (i.e., the alveus) without involving synaptic transmissions.

### No spreading depression but more attenuation of APS amplitudes caused by A-HFS of monophasic pulses

**Fig. 4:**
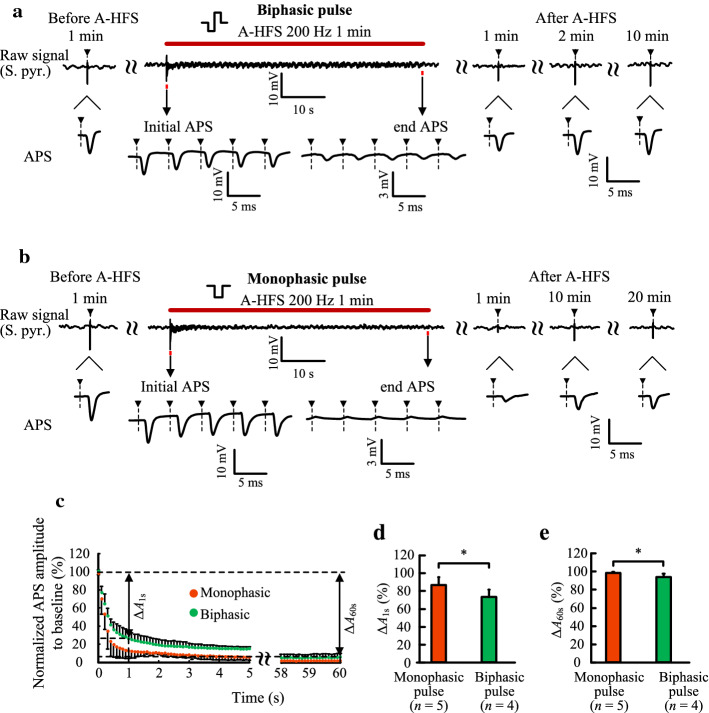
Neuronal responses induced by 200-Hz A-HFS in the hippocampal CA1 region. **a** and **b** Top*:* typical examples of recording signals in the S. pyr. with 1-min 200-Hz A-HFS (denoted by the red bar) of biphasic pulses (**a**) or monophasic pulses (**b**). Bottom: typical waveforms of APS induced by A-test pulses or A-HFS pulses in the periods before, during and after A-HFS. **c** Normalized APS amplitude in the first 5 s and last 2 s of 1-min A-HFS. **d** Comparison of the decrement of APS amplitude at 1 s (Δ*A*_1s_) between monophasic A-HFS (*n* = 5) and biphasic A-HFS (*n* = 4). **e** Comparison of the decrement of APS amplitude at 60 s (Δ*A*_60s_) between monophasic A-HFS (*n* = 5) and biphasic A-HFS (*n* = 4). ^*^P < 0.05, *t*-test

To investigate the neuronal responses to the A-HFS of biphasic and monophasic pulses, we analyzed each APS evoked by each pulse of A-HFS. During the 1-min 200-Hz A-HFS of biphasic pulses, APS was able to follow each stimulation pulse. However, the APS waveforms with a large amplitude only appeared at the initial period. The APS amplitudes decreased rapidly with the proceeding of A-HFS (Fig. 4a), which may be caused by the depolarization block of axons [5, 20]. After the end of A-HFS, the test APS recovered to ~ 70% of baseline level in ~ 1 min and to 92.2 ± 21.0% (*n* = 4) of baseline level in ~ 2 min.

When the A-HFS was applied with monophasic pulses, the neuronal responses at the initial period were similar to that of biphasic pulses—each pulse induced a large APS. However, at the late period of A-HFS, the monophasic pulses hardly induced APS (Fig. 4b). In addition, after the end of A-HFS, the test APS only recovered to 34.5 ± 12.1% (*n* = 5) of baseline level even after ~ 20 min.

To compare the attenuations of APS during the A-HFS with biphasic and monophasic pulses, we calculated the APS amplitudes normalized to baseline value to evaluate the changes of APS (Fig. 4c). The decrement of APS amplitudes at the initial 1 s (Δ*A*_1s_) of A-HFS was significantly greater with monophasic A-HFS (86.9 ± 8.7%, *n* = 5) than with biphasic A-HFS (73.5 ± 8.0%, *n* = 4; *t*-test, *P* < 0.05. Fig. 4d). Also, the decrement of APS amplitudes at the end of A-HFS (Δ*A*_60s_) was significantly greater with monophasic A-HFS (98.6 ± 1.2%, *n* = 5) than with biphasic A-HFS (94.1 ± 3.4%, *n* = 4; *t*-test, *P* < 0.05. Fig. 4e).

The faster and larger attenuation of APS amplitudes during monophasic A-HFS indicated that the monophasic pulses can cause more conduction failures than biphasic pulses. The partial recovery of test APS after monophasic A-HFS indicated that the conduction failures in at least a portion of axons can even persist after the A-HFS.

In addition, no SD event was observed in all the 9 rats applied 1-min 200-Hz A-HFS (4 with biphasic pulses and 5 with monophasic pulses). That is, the SD incidence during 200-Hz monophasic A-HFS (0/5) was significantly smaller than that during 200-Hz monophasic O-HFS (4/5; Fisher’s exact test, *P* < 0.05).

## Discussion

The major findings of the present study in the rat hippocampal CA1 region include: O-HFS of monophasic pulses at the afferent fibers with an enough high frequency can induce spreading depression (SD) events and affect the pathway of orthodromic activation; and A-HFS of monophasic pulses at the efferent fibers can cause conduction failures in a portion of axons even after the A-HFS. Possible mechanisms underlying the findings are discussed below.

SD is a type of large shift of depolarization potential in brain regions. The earliest discovery of SD was associated with epileptic bursts and was described as a suppression of electroencephalographic signals following bursts of seizure discharges in the cortex regions [21]. Studies have also shown that SD events can follow neuronal discharges in other brain regions, such as in hippocampus [18]. One of the major associated characteristics of SD is the great concentration changes of certain ions in the extracellular space, especially a substantial elevation of extracellular potassium concentration ([K^+^]_o_), indicating redistributions of the ions between extra- and intra-cellular spaces [22].

In the present study, SD events only appeared during monophasic O-HFS but not during biphasic O-HFS. Thus, certain effects associated with the HFS of monophasic pulses could be responsible for the appearance of SD. In contrary to the biphasic pulses, the monophasic pulses drive the ions in fixed directions in the brain tissue by the force of the unidirectional electric field. The cations migrate to the cathodic pole of the electrode and the anions to the anodic one. Under the action of repetitive monophasic pulses with a high frequency, the ions may accumulate around the two poles of the electrode and destroy the ionic equilibriums between the intracellular and extracellular environments. Previous studies have shown that repetitive stimulations can enhance [K^+^]_o_ in the CA1 region [23]. Presumably, the increase of [K^+^]_o_ may be exacerbated by the HFS of monophasic pulses because of the cationic accumulation by the unidirectional driving force around the small space of electrode poles (Fig. 1b). The high [K^+^]_o_ may result in a depolarization in neuronal membranes and generate epileptiform activity [24, 25], just as the burst of population spikes preceding SD trough in the present study (Fig. 3b). The burst activity may further elevate [K^+^]_o_ thereby resulting in the large shift of depolarization potential of SD. Given the fact that a potassium injection with a molar level of concentration can induce SD events in animal preparations [14, 26, 27], the accumulation of K^+^ by monophasic pulses may be one of the mechanisms to generate SD.

In addition, synapse-related effects could also contribute to the generation of SD, because SD events only appeared during O-HFS involving synaptic transmissions but did not appear during A-HFS. Previous studies have shown that an excessive concentration of glutamates released from excitatory synapses to the extracellular space can induce SD [27–29]. In the present study, the O-HFS in the afferent fibers can activate the excitatory synapses and can cause glutamates releases at the layer of apical dendrites of pyramidal neurons (i.e., S. rad.) that is full of glutamatergic synapses. In addition, the elevation of [K^+^]_o_ by monophasic O-HFS may reduce the uptake of glutamates and reduce extracellular space by astrocytes swelling, thereby further increasing the concentration of extracellular glutamates [30, 31]. Thus, an elevation of glutamates caused by monophasic O-HFS can be another factor to generate SD.

Furthermore, the non-recovery of the test OPS after monophasic O-HFS and the partial recovery of APS after monophasic A-HFS suggested that the application of intensive monophasic pulses could have damaged the axons under the stimulations. As previously reported, the toxic chemical products of irreversible chemical reactions at the electrode–tissue interface generated by monophasic stimulations can damage the neuronal tissues [8]. The reactions include electrolysis of water, oxidation of saline, metal, and organic materials, as well as reduction of oxygen [32, 33]. In addition, according to mass action theory, an extreme excitation by over-stimulating the excitable tissue can result in the depletion of oxygen or/and glucose thereby substantially changing the ionic concentrations in both intracellular and extracellular regions [8]. These factors may result in neuronal dysfunction and tissue damages. Further histological and morphological analyses of stimulated tissues are needed to confirm the tissue damages. Nevertheless, the appearance of SD can act as an index to warn the tissue damages.

Taken together, an extreme elevation of [K^+^]_o_ and an increase of glutamate concentrations can result in the generation of SD by the monophasic O-HFS. In addition, the irreversible chemical reactions induced by the monophasic HFS with an enough high frequency could damage a portion of the axons in the stimulation site.

## Conclusions

Our results showed that orthodromically activating hippocampal neurons by HFS of monophasic pulses can induce the abnormal neuron activity of spreading depression, and antidromic activation can cause an attenuation of neuronal excitability persisting after HFS stimulations. The results suggest that HFS of monophasic pulses have a risk of tissue damage and can induce neuronal reactions different from HFS of biphasic pulses, which provide new information for appropriate usage of pulse stimulations in brain tissues either in clinic or in animal experiments.

## Methods

### Surgical procedures

The animal experiment was approved by the Institutional Animal Care and Use Committee, Zhejiang University. Twenty adult male Sprague-Dawley rats were used: 11 for O-HFS (6 for biphasic pulses and 5 for monophasic pulses) and 9 for A-HFS (4 for biphasic pulses and 5 for monophasic pulses). Each rat was anesthetized with urethane (1.25 g/kg, i.p.) and was placed in a stereotaxic apparatus (Stoelting Co.). Details of the surgery and the electrode placements were similar to the previous report [34]. Briefly, a 16-channel recording electrode array (#A4 × 4-3 mm 50–125–177, NeuroNexus Technologies, USA) was inserted into the hippocampal CA1 region to record electrical potentials. Two concentric bipolar stimulation electrodes (#CBBRC75, FHC Inc., USA) were positioned in the Schaffer collaterals and the alveus fibers of CA1 region for orthodromic- and antidromic-HFS, respectively (Fig. 1a). The distance between the inner and outer poles of the stimulation electrodes was short (< 100 μm) (Fig. 1b), indicating a small area around the two poles. The signals of multiple unit activity (MUA) and the waveforms of both orthodromically evoked population spike (OPS) and antidromically evoked population spike (APS) along the recording channels were used to guide the final positions of the three electrodes.

### Stimulation and recording

Sequences of current pulses of monophase (negative phase) and biphase (a preceding negative phase followed by a positive phase) with a phase width of 0.1 ms (Fig. 1c) were generated by a stimulator (Model 3800, A-M Systems Inc., USA). The current intensity was 0.3–0.5 mA that was able to induce a population spike (PS) with an amplitude approximately 75% of the maximal PS amplitude. The pulse frequency was 100 or 200 Hz for O-HFS at the Schaffer collaterals, and 200 Hz for A-HFS at the alveus fibers of the hippocampal CA1 region. The duration was 1 min for both O- and A-HFS.

Recording signals were amplified 100 times by an AC-coupled (0.3–5000 Hz) amplifier (Model 3600, A-M Systems Inc., USA). The amplified signals were then sampled at a rate of 20 kHz by a Powerlab data acquisition system (ADInstruments Inc., Australia).

### Data analysis

The artifacts of stimulation pulses in the raw recording signals were removed by an algorithm to detect each artifact segment (~ 1.0 ms) that was then replaced by an interpolation line [35]. The artifact-free signals were then filtered by high-pass digital filters with a cut-off frequency of 500 Hz to obtain the MUA signals, or with a cut-off frequency of 10 Hz to remove the slow waveform of SD to illustrate the burst population spikes accompanying SD. The recording signals from the channel closest to the cell body layer (i.e., S. pyr.) were used to calculate the amplitudes of evoked-PS.

Data were expressed as mean ± standard deviation and *n* represented the number of rat experiments. Statistical Fisher’s exact test and *t*-test were used to judge the significance of differences among data groups.

## Data Availability

All data generated or analyzed during this study are included in this published article.

## Abbreviations

DBS: Deep brain stimulation
HFS: High-frequency stimulation
SD: Spreading depression
OPS: Orthodromically evoked population spikes
APS: Antidromically evoked population spikes
MUA: Multiple unit activity
S. rad.: Stratum radiatum
S. pyr.: Stratum pyramidale
S. ori.: Stratum oriens
